# Odor hedonics coding in the vertebrate olfactory bulb

**DOI:** 10.1007/s00441-020-03372-w

**Published:** 2021-01-30

**Authors:** Florence Kermen, Nathalie Mandairon, Laura Chalençon

**Affiliations:** 1 Department of Biology, Faculty of Natural Sciences, Norwegian University of Science and Technology, 7030 Trondheim, Norway; 2 CNRS. UMR 5292: INSERM, U1028: Lyon Neuroscience Research Center Neuroplasticity and Neuropathology of Olfactory Perception Team, University Lyon, University Lyon1, F-69000 Villeurbanne, France

**Keywords:** Olfactory bulb, Hedonic value, Innate and learned valence, Odor preference, Behavior, Mouse, Rat, Zebrafish, Human

## Abstract

Whether an odorant is perceived as pleasant or unpleasant (hedonic value) governs a range of crucial behaviors: foraging, escaping danger, and social interaction. Despite its importance in olfactory perception, little is known regarding how odor hedonics is represented and encoded in the brain. Here, we review recent findings describing how odorant hedonic value is represented in the first olfaction processing center, the olfactory bulb. We discuss how olfactory bulb circuits might contribute to the coding of innate and learned odorant hedonics in addition to the odorant’s physicochemical properties.

## Introduction

When we smell an odorant, our reaction is often that “I like it” or “I don’t like it” (Richardson and Zucco 1989). This is known as odor hedonic value. A pleasant odorant has a positive hedonic value and may be predictive of a reward, whereas an unpleasant odorant has a negative hedonic value and may be predictive of a punishment. Odor hedonics can be unconditioned (innate) or learned and largely dominates olfactory perception. In addition to being the first aspect used to describe and categorize odors (Berglund et al. 1973; Schiffman et al. 1977), odor hedonic value is the most discriminating dimension in multidimensional analyses of odor verbal descriptors (Khan et al. 2007; Moskowitz and Barbe 1977; Zarzo 2008).

Odor hedonics is also the foundation of olfactory pleasure. Hence, deterioration in odor hedonic value in normal aging (Joussain et al. 2013), parosmia (Walliczek-Dworschak and Hummel 2017) or neurodegenerative (Joussain et al. 2015; Mrochen et al. 2016) and neuropsychiatric conditions (Atanasova et al. 2010; Lombion-Pouthier et al. 2006; Moberg et al. 2003; Naudin et al. 2014; Walsh-Messinger et al. 2018) impairs the wellbeing of a significant number of the population. More generally, odor hedonic value governs approach/avoidance behavior in most vertebrates, crucially contributing to behaviors that are important for fitness and survival, such as feeding, social interaction and predator avoidance.

Odor hedonic value can be measured directly using subjective questionnaires in humans (Ferdenzi et al. 2016; Khan et al. 2007; Mandairon et al. 2009; Zarzo 2008) and/or indirectly by assessing odor-evoked autonomic responses (Alaoui-Ismaïli et al. 1997; Bensafi et al. 2002; Brauchli et al. 1995). In other vertebrates, odor hedonics is most commonly inferred from odorant investigation time (*rodents* (Jagetia et al. 2018; Kermen et al. 2016; Kobayakawa et al. 2007; Mandairon et al. 2009; Saraiva et al. 2016); *fish* (Hussain et al. 2013)), or from metrics quantifying species-specific appetitive and defensive behaviors (Frank et al. 2019; Kermen et al. 2020a; Mathuru et al. 2012; Yabuki et al. 2016). Despite some interindividual variability in odor hedonics in humans (Rouby et al. 2009), mice (Jagetia et al. 2018), and zebrafish (Kermen et al. 2020a), these psychophysical and behavioral approaches enabled species-specific odor preference gradients to be established at population level: from odorants with positive hedonic value (attractive, such as food odor) to neutral and negative ones (aversive, such as rotten food, which is avoided), or danger signals (predator odor, which induces a panic response). Interestingly, odor preferences might be partially conserved among some vertebrates; for instance, odor investigation times in mice are positively correlated with pleasantness ratings in humans (Mandairon et al. 2009) (but see (Manoel et al. 2019))*.*

Given that odor hedonics is such a prominent aspect of olfactory perception in a broad range of species, abundant research has focused on understanding the neural underpinnings in the brain*.* Early research found neural representation of odor hedonics in the orbitofrontal cortex, insula, amygdala, and piriform cortex (Bensafi et al. 2007; Grabenhorst et al. 2007; Katata et al. 2009; Winston et al. 2005). More recently, a number of studies in human and animal models converged in showing that representations of odor hedonic value can be found at all levels of the olfactory system (reviewed in (Mantel et al. 2019)), including early levels, the olfactory epithelium (Lapid et al. 2011), and the olfactory bulb (OB) (Doucette et al. 2011; Kay and Laurent 1999; Kermen et al. 2016; Kobayakawa et al. 2007; Haddad et al. 2010).

In this review, we discuss how odor hedonics is represented and processed by the OB circuits. After briefly introducing the anatomical and functional features of the OB circuits, we discuss (1) a possible topographic OB representation of innate odor hedonic value, (2) how OB circuits represent and support learned odorant hedonic value, (3) how innate and learned odor hedonic values are integrated in OB circuits, and (4) the contribution of the OB in processing complex signals composed of odorants with different hedonic values.

## Olfactory bulb odor maps and plasticity

In vertebrates, information from olfactory sensory neurons (OSNs) located in the nose converges onto the OB. There, OSN axons contact OB output neurons—mitral/tufted cells in rodents and mitral/ruffled cells in fish—and local modulatory interneurons, within functional processing units called glomeruli (Nagayama et al. 2014; Satou 1990). As a result, odors are represented in the OB by odorant-specific spatiotemporal maps that are relatively consistent across individuals of the same species (Baier and Korsching 1994; Rubin and Katz 1999). These glomerular activity patterns are modulated by functionally diverse populations of pre- and post-synaptic OB interneurons. Periglomerular interneurons mediate interglomerular interactions, enabling olfactory contrast enhancement and input decorrelation (Cleland and Sethupathy 2006; Wanner and Friedrich 2020). Deeper in the OB, the odor pattern is regulated by feedback loops between output neurons and granule cells that are involved in olfactory discrimination and memory (Grelat et al. 2018; Mori and Sakano 2011; Mori and Yoshihara 1995; Tan et al. 2010). Interestingly, approximately half of the bulbar interneurons are regenerated throughout life by adult neurogenesis, conferring additional plasticity to the system (Altman 1969; Imayoshi et al. 2008; Lledo and Valley 2016). In addition, bulbar odor activity patterns are further modulated via top-down input from brain regions involved in arousal, learning, and hedonics (Linster and Devore 2012; Padmanabhan et al. 2019).

The function of spatially organized OB activity maps and how they guide odor perception remain unclear. A coarse chemotopic organization is observed at local scale in zebrafish (Friedrich and Korsching 1998) and rodents (Chae et al. 2019; Rubin and Katz 1999). OB input and output patterns only partially relate to the odorants’ physicochemical properties, which suggests they may also represent contextually and behaviorally relevant information, including fine odor discrimination (Linster et al. 2001) and odor hedonics (Chae et al. 2019; Haddad et al. 2010). Hence, both the topographical organization of odor maps and the wide range of neural computations performed by OB circuits are able to represent and impart meaning to an odor according to species-specific evolutionary constraints, olfactory context and past experience, before it is passed on to higher brain centers.

## Is there a topographic organization of innate odorant hedonics in the olfactory bulb?

**Fig. 1:**
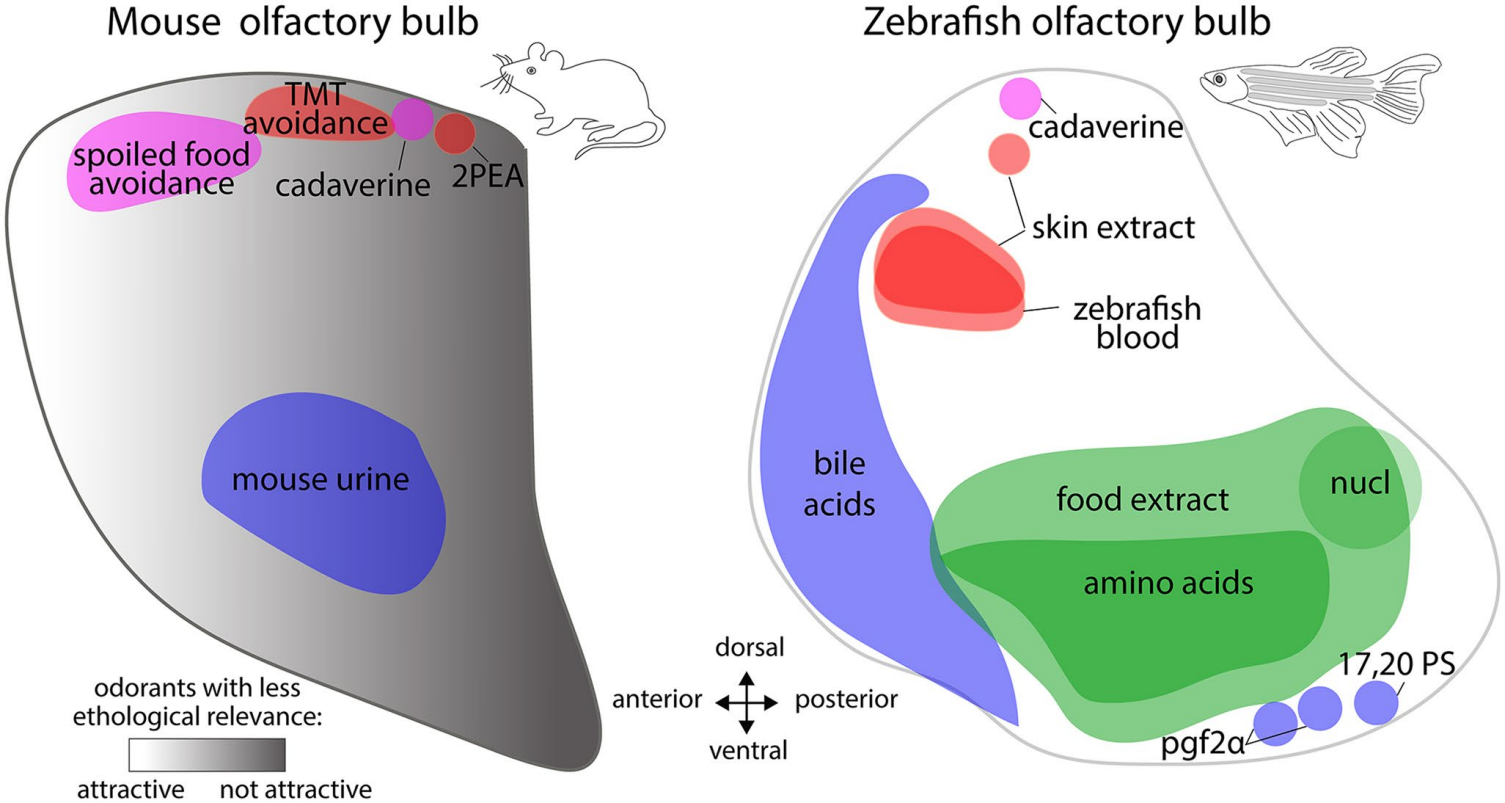
Spatial olfactory bulb domains activated by aversive and appetitive odorants in the mouse and the zebrafish Colored areas correspond to the olfactory bulb domain either responding to an odorant, or mediating the behavioral response to that odorant. Red areas represent domains responding to predator odors. Magenta areas represent domains responding to aversive spoiled food or decaying flesh. Green areas represent domains responding to food odors. Blues areas represent domains responding to putative social cues released by conspecifics. These maps are based on previously published works that are listed in Table 1. In the mouse olfactory bulb, the grayscale gradient represents zones of the granule cell layer that are preferentially activated by attractive versus unattractive odors (Kermen et al. 2016). Note that on this lateral view of the olfactory bulb, no distinction is made between domains located in the medial and lateral halves. nucl, nucleotides; TMT, 2,3,5-trimethyl-3-thiazoline; 2PEA, 2-phenylethylamine; pgf2α, prostaglandin F2α; 17,20 PS, 17alpha, 20beta-dihydroxy-4-pregnene-3-one-20-sulfate

In the fruit fly’s antennal lobe, aversive odorants activate output neurons innervating glomeruli located more medially than those recruited by attractive odorants (Knaden et al. 2012; Seki et al. 2017). Hence, the hedonic value of an odorant is first represented in the antennal lobe by spatially segregated groups of glomeruli. A similar segregated arrangement of hedonic channels could also be present to some extent in the vertebrate OB (Fig. 1; Table 1).

The ventral domain of the vertebrate OB may be specialized in detecting appetitive and social odors. In the zebrafish, appetitive food-derived odors and attractive sex pheromones activate the ventrolateral and ventromedial OB, respectively (Kermen et al. 2020b; Yabuki et al. 2016; Yoshihara 2014). Mice of both sexes are strongly attracted to the odor of opposite-sex urine, which activates mitral cells located in the ventral OB (Kang et al. 2009; Xu et al. 2005). In rats, systematic analysis of hundreds of 2-deoxyglucose glomerular activation patterns revealed that floral, woody, fruity and herbaceous odorants, which are rated as pleasant by humans, preferentially activate the rat ventral OB (Auffarth et al. 2011).

In contrast, the dorsal OB, although it also responds to a large number of odorants with neutral hedonic value, plays a specific role in the processing of odorants signaling danger. The OSNs expressing trace amine-associated receptors, which mediate detection of spoiled flesh and/or predator odorants, project onto the dorsal OB in both zebrafish and mice (Dieris et al. 2017; Hussain et al. 2013; Pacifico et al. 2012). In the zebrafish, aversive odorants signaling decaying flesh and fear-inducing alarm odorants mostly activate the dorsal and dorsolateral OB domains, respectively (Dieris et al. 2017; Kermen et al. 2020a; Mathuru et al. 2012; Yoshihara 2014). Although spoiled food and predator odorants activate multiple glomeruli in both ventral and dorsal OB domains in rodents (Kobayakawa et al. 2007), the innate response to these odorants seems to rely solely on information conveyed by the dorsal domain. Optogenetic activation of a posterodorsal glomerulus responding to 2,3,5-trimethyl-3-thiazoline (TMT; a component of fox odor) is sufficient to induce freezing (Saito et al. 2017), whereas disrupting the function of dorsal TMT-responsive glomeruli impairs TMT-induced aversive behavior (Cho et al. 2011; Saito et al. 2017). Additionally, mutant mice devoid of dorsal glomeruli can detect the smell of spoiled food or predators, but do not display the same innate aversive response to these odors as their wild-type conspecifics (Kobayakawa et al. 2007)*.*

In line with the role of the dorsal OB in mediating responses to aversive odorants with strong ethological relevance, unpleasant odorants described by human as “medicinal” (wintergreen, eucalyptus) or reminiscent of detergents strongly activate the dorsal OB in rats (Auffarth et al. 2011). Another study in mice showed that unlearned unattractive odorants with no particular ethological relevance activate the posterodorsal part of the glomerular layer to a greater extent than unlearned attractive odorants (Kermen et al. 2016). Furthermore, this study revealed an additional level of OB functional organization, in which odor hedonic information is represented along the anteroposterior axis of the ventral OB. The authors showed that unpleasant odorants evoked greater activity in the posteroventral OB, whereas pleasant odorants evoked greater activity in the anteroventral area. Manipulation of this OB hedonic signature using optogenetics reverted the initial odorant preference (Kermen et al. 2016).

Are these segregated hedonic OB representations preserved in the projections to higher brain centers? In mice, depending on their anteroposterior location in the OB, mitral cells differentially target the olfactory tubercle (Imamura et al. 2011; Midroit et al. 2020), a brain region known to code odor hedonic value (Gadziola et al. 2015; Midroit et al. 2020). The cortical amygdala, which is a brain region involved in innate olfactory behavior (Root et al. 2014), primarily receives dorsal OB input (Miyamichi et al. 2011). Moreover, the anterior olfactory nucleus receives topographically organized projections from the dorsoventral axis of the OB (Miyamichi et al. 2011). These topographically organized cortical projections suggest that certain cortical OB targets might utilize the spatial segregation of hedonic information within the OB.

Taken together, the above findings suggest that the vertebrate OB is coarsely organized into nested axes that differentially mediate behavioral response to odorants, depending on hedonic value and ethological relevance. It would be interesting to determine whether these hedonic axes are conserved in the OB of other species than mice, rats and zebrafish, reflecting a general organizational principle in vertebrate olfaction. Additional systematic studies of downstream OB projection patterns would help in understanding how OB hedonic channels are distributed toward higher brain regions.

**Table 1:** List of odorants and references used in Fig. 1

Odorants	Odor category	Species	References
Cadaverine	Aversive decay odor	Zebrafish	(Dieris et al. 2017)
Zebrafish blood	Alarm odor	Zebrafish	(Kermen et al. 2020a)
Zebrafish skin extract	Alarm cue	Zebrafish	(Chia et al. 2019; Diaz-Verdugo et al. 2019; Kermen et al. 2020a; Mathuru et al. 2012; Yoshihara 2014)
Amino acids	Attractive food odor	Zebrafish	(Friedrich and Korsching 1998; Koide et al. 2009; Tabor et al. 2004; Yaksi et al. 2009)
Food extract	Attractive food odor	Zebrafish	(Kermen et al. 2020b,a; Tabor et al. 2004)
Nucleotides	Attractive food odor		(Friedrich and Korsching 1998; Wakisaka et al. 2017)
Pgf2α; 17,20 PS	Reproductive pheromones	Zebrafish	(Dieris et al. 2017; Friedrich and Korsching 1998; Kermen et al. 2020b; Yabuki et al. 2016)
Bile acids	Putative social odor	Zebrafish	(Friedrich and Korsching 1998; Kermen et al. 2020a; Koide et al. 2009; Yaksi et al. 2009)
TMT	Predator odor	Mouse	(Cho et al. 2011; Igarashi et al. 2012; Kobayakawa et al. 2007; Saito et al. 2017)
2PEA	Predator odor	Mouse	(Ferrero et al. 2011)
Mouse urine	Attractive conspecific odor	Mouse	(Kang et al. 2009; Martel and Baum 2007; Martel et al. 2007; Schaefer et al. 2001; Xu et al. 2005)
Pentanal; 2-methylbutyric acid; alkylamines	Spoiled food	Mouse	(Igarashi et al. 2012; Kobayakawa et al. 2007; Takahashi et al. 2004)

Note that the list of bibliographic references used to map the OB domain activated by a given odorant is not exhaustive. TMT, 2,3,5-trimethyl-3-thiazoline; 2PEA, 2-phenylethylamine; pgf2α, prostaglandin F2α; 17,20 PS, 17alpha, 20beta-dihydroxy-4-pregnene-3-one-20-sulfate

## Representation of learned hedonic value in olfactory bulb circuits

How does the OB network contribute to learned odor hedonics? The general contribution of OB circuits to different types of olfactory learning (habituation, perceptual, and associative learning) has been extensively reviewed elsewhere (Lledo and Valley 2016; Ross and Fletcher 2019; Tong et al. 2014; Wilson and Sullivan 1994). Here, we specifically discuss how bulbar activity patterns are modified when odorant hedonic value switches from neutral to appetitive or aversive, and the underlying mechanisms.

Early evidence, by unit recordings in the OB of anesthetized and awake mice performing an associative learning task, indicated that mitral cell firing rate is influenced by conditioned odor hedonic value (Doucette et al. 2011; Kay and Laurent 1999). Both appetitive and fear associative learnings durably modify behavioral response and odorant representation in the OB (at input, and possibly in the output layers) (Coopersmith et al. 1986; Fletcher 2012; Kass and McGann 2017; Sullivan and Leon 1987). Interestingly, fear learning—compared to appetitive learning—alters the animal’s defensive responses and the bulbar network in a way that is not odorant specific. Animals conditioned by foot shock developed a generalized fear response, not only to the learned odorants but also to odorants that were structurally unrelated (Kass and McGann 2017; Ross and Fletcher 2018). Paralleling this, olfactory fear learning enhances not only the representation of the learned odorant but also that of the unconditioned odorants in periglomerular interneurons (Kass and McGann 2017) and output neurons (Ross and Fletcher 2018).

The OB network has a specificity in rodents: it is the target of adult neurogenesis that has been shown to be involved in different types of olfactory learning. Adult neurogenesis underlies the acquisition and/or memory of associative appetitive learning (Alonso et al. 2012; Kermen et al. 2010; Mandairon et al. 2011; Mouret et al. 2009; Sultan et al. 2010) and fear conditioning (Valley et al. 2009). While a large number of studies focused on how adult-born neurons support olfactory learning, it remains unclear whether adult OB neurogenesis is involved in the acquisition of a new odor hedonic value after conditioning.

## Different olfactory bulb neuronal populations support innate and learned hedonic value, respectively

How are innate and learned odor hedonics represented in OB circuits? Recent studies indicate that innate responses to odorants signaling danger and learned appetitive responses to novel odorants might be mediated by developmentally distinct populations of OB neurons. Innate aversion to predator odorants appears to depend on OB circuits formed in early development, around birth. In adult mice, fear response to the predator odor TMT was abolished by inactivation of subsets of perinatally born, but not adult-born, olfactory neurons (Muthusamy et al. 2017; Sakamoto et al. 2011). Perinatal exposure to aversive odorants disturbs aversion behavior later in life, which indicates that innately aversive odorants exhibit a very restricted window of perinatal plasticity (Qiu et al. 2020a), after which their hedonic value cannot be altered. In contrast, detection of novel odorants paired with a positive reward is resilient to early-born neuron silencing, but impaired by inactivation of subpopulations of adult-born granule cells (Muthusamy et al. 2017). Similar lines of evidence show that the activity of adult-born but not perinatally born granule cells contains information about an odor’s learned positive hedonic value (Grelat et al. 2018). This differential involvement of early- and late-born neurons in learned odor hedonics could be explained by the fact that late-born but not early-born granule cells display a rapid form of structural plasticity (Breton-Provencher et al. 2016).

Taken together, these studies support the idea that innate (predator) odorants are processed via hardwired circuits, established during early life stages and with a narrow window of plasticity, whereas the appetitive learning of novel odors is flexibly supported throughout life via remodeling of OB response by plastic adult-born neurons.

## Odor hedonics in realistic conditions: olfactory bulb processing of hedonically complex odor blends

In natural situations, odors often consist of hedonically complex sensory signals comprising both positive and negative hedonic components. In order to survive, animals need to make rapid behavioral decisions based on these conflicting sensory inputs. For example, it might be evolutionarily advantageous to detect the odor of a predator even in presence of appetitive feeding or reproductive olfactory cues. Despite a few attempts at predicting the hedonic value of odorant mixtures in humans (reviewed in Thomas-Danguin et al. (2014)), surprisingly little is known regarding the integration of ethologically relevant odorants with contrasting hedonic values in the OB circuits.

The neural representation of complex odor blends at different stages of the olfactory system primarily involves component suppression, which can result in a new percept, qualitatively different from the individual components, or in the dominance of one odorant over the others (Thomas-Danguin et al. 2014)*.* Although suppressive interactions between odorants can arise from peripheral effects in the nose, a large part originates from local OB circuit computations (Economo et al. 2016; Linster and Cleland 2004; Qiu et al. 2020b; Tabor et al. 2004). In the OB, interactions have been documented between odorants of similar or opposite hedonic value. The odor of fennel or clove, which are spices commonly used by humans to mask the flavor of spoiled food, suppresses the activity of spoiled food odorant-responsive mitral cells in mice (Takahashi et al. 2004). In the zebrafish, appetitive food extracts suppress mitral cell response to an attractive reproductive pheromone (Kermen et al. 2020b), which indicates that OB circuits might favor the detection of sensory cues relevant to immediate rather than delayed physiological needs. Interestingly, odorants with negative hedonic value, such as the predator odorants TMT and 2-phenylethylamine (a component of bobcat urine which is avoided by rats and mice (Ferrero et al. 2011)), systematically block or override mice’s behavioral attraction to appetitive odorants, and result instead in neutral or avoidance responses (Saraiva et al. 2016). In addition, when predator odorants are presented as a target stimulus atop a continuous neutral hedonic odor background, the resulting OB and behavioral responses are highly correlated to those elicited by the predator odorants alone (Qiu et al. 2020b). This indicates that predator odorants are relatively well protected against suppression by other odorants and dominate behavioral outcome. Since OB representations of target odorants without ethological relevance can on some occasions be completely masked by the background (Vinograd et al. 2017), one hypothesis is that the animal’s ability to detect an odorant within an olfactory background depends on the hedonic value of the target odorant. Whether the representation of odorants with negative hedonic value is specifically preserved in a noisy olfactory context, or if it is rather a general feature of salient odorants important for survival (Vinograd et al. 2017), remains unknown and is an exciting area for future research.

Studying the organization of interglomerular inhibitory circuits (i.e., the periglomerular and short axon cell networks), which are thought to mediate suppressive interactions between odorants, could shed light on how the OB integrates hedonically complex odors. Recent anatomical and functional evidence shows that interglomerular inhibition, far from being randomly organized, is odor and/or glomerulus specific in zebrafish (Wanner et al. 2016) and mouse (Economo et al. 2016) OBs, which could favor inhibition between processing channels with different hedonic value. In particular, some murine glomeruli are suppressed by a large number of odorants, whereas other glomeruli are particularly resilient to suppression (Economo et al. 2016). Studies investigating the odor tuning of resilient and suppressed glomeruli with respect to ethological relevant stimuli would help to determine whether interglomerular suppression contributes to the representation of hedonically complex signals, as reported in the fruit-fly’s antennal lobe (Berck et al. 2016; Mohamed et al. 2019).

Taken together, these studies show that nonlinear interactions within OB circuits between components of hedonically complex odor signals can support biased behavioral response to a specific component. Nonlinear interactions in the vertebrate OB may thus play an important and underexplored role in hedonic perception in natural conditions.

## Conclusion

Several representations of odor hedonics are present in the OB and seem to recruit different circuit mechanisms and/or topographical locations according to whether the odorant is ethologically relevant or not and whether the hedonic value was innate or learned. Overall, these studies critically demonstrate the crucial contribution of OB circuits in attributing hedonic value to odorants throughout life. To provide a better understanding of these differences, future studies should focus on the comparison between innate hedonic value of odorants with or without biological significance and learned odor hedonic value.
